# A82 A SURVEY-BASED ASSESSMENT OF RADIATION PROTECTION ATTITUDES AND PRACTICES IN ADVANCED THERAPEUTIC ENDOSCOPY TRAINING CURRICULUMS

**DOI:** 10.1093/jcag/gwad061.082

**Published:** 2024-02-14

**Authors:** K Chin Koon Siw, C Sypkes, M Gozdzik, H Dhaliwal, A Kundra

**Affiliations:** Division of Gastroenterology and Hepatology, University of Ottawa, Ottawa, ON, Canada; Division of Internal Medicine, University of British Columbia, Vancouver, BC, Canada; Division of Gastroenterology, University of Alberta, Edmonton, AB, Canada; Division of Gastroenterology and Hepatology, University of Ottawa, Ottawa, ON, Canada; Division of Gastroenterology and Hepatology, University of Virginia Health Services, Charlottesville, VA

## Abstract

**Background:**

Fluoroscopy-assisted endoscopic procedures such as endoscopic retrograde cholangiopancreatography (ERCP) expose patients and physicians to the risks of ionizing radiation, including infertility, cataracts, skin hypersensitivity, hair loss and cancer. Radiation exposure from these procedures can have lasting adverse effects on the health and productivity of gastroenterologists. With attention to “as low as reasonably achievable” (ALARA) principles, recent efforts to minimize radiation use have emphasized the importance of formal radiation protection education, including during advanced therapeutic endoscopy (ATE) training. However, teaching of basic protective measures in radiation safety during ATE training remains often overlooked.

**Aims:**

We aimed to learn how radiation safety training is delivered in ATE programs across Canada and the US. Identifying the existing training standards and knowledge will help us understand the current gaps in training curriculums and inform future curricular development.

**Methods:**

Our study was reviewed by the Ottawa Health Science Network Research Ethics Board in September 2023. Surveys were designed and distributed with the REDCap software and sent to ATE trainees (current and recent graduates within the last 3 years) in Canada and Program Directors (PDs) of accredited ATE programs across Canada and the US.

**Results:**

The survey was completed by 12 trainees and 4 PDs. These are preliminary results and further survey responses are being collected. The majority of trainees (75%) believed it would be very important for their program’s curriculum to include dedicated radiation safety teaching, but most trainees (83.3%) received no such training. Familiarity in optimizing modifiable fluoroscopy parameters to minimize radiation exposure (collimation, gain, magnification, frame rate modification or pulsed fluoroscopy) was variable among trainees (36.4%-81.8%). Seventy-five percent of trainees were unaware of the annual limit for radiation exposure and only 25% believed they were operating within a safe level. Most trainees (90.9%) felt they were given inadequate training to optimally reduce their risk of radiation exposure and 90.9% expressed a desire for further radiation safety training. Despite these findings, only 50% of PDs believed it would be beneficial to offer dedicated radiation safety training as part of their program’s curriculum. Among PDs, 75% did not feel their trainees had the knowledge required to reduce their risk of radiation exposure and all (100%) felt that dedicated training would be useful.

**Conclusions:**

These findings indicate a need for more robust radiation safety training. Further studies are needed to understand the most effective way to deliver comprehensive radiation safety training to ATE trainees.

**Funding Agencies:**

None

